# Assessment of factors affecting tourism satisfaction using K-nearest neighborhood and random forest models

**DOI:** 10.1186/s13104-019-4799-6

**Published:** 2019-11-19

**Authors:** Leili Tapak, Hamed Abbasi, Hamid Mirhashemi

**Affiliations:** 1 Department of Biostatistics, School of Public Health, Hamadan University of Medical Sciences, Hamadan, 65175-4171 Iran; 2 Modeling of Noncommunicable Diseases Research Center, Hamadan University of Medical Sciences, Hamadan, Iran; 3 Department of Geography, Lorestan University, Lorestan, Iran

**Keywords:** Tourism, Satisfaction, Random forest, K-nearest-neighborhood

## Abstract

**Objective:**

This study aimed to identify factors affecting the satisfaction of tourists traveling to the city of Hamadan as Asian urban tourism capital in 2018. The data a random sample of 300 tourists were collected using a designed questionnaire. We applied random-forest and K-nearest-neighborhood methods to analyze the data.

**Results:**

The variables of society behavior, municipal equipment and cost of services were the three top rank variables in predicting tourist satisfaction. Considering the capacity of the ancient city of Hamadan for tourism, policy-makers can use our results in planning for providing a sustainable development and flourishing tourism industry in this city.

## Introduction

Currently, tourism is one of the most dynamic economic activities with many socio-economic/environmental/cultural benefits. Nowadays, economists call it invisible exports. Tourism industry involves the presence of different economic sectors, so, the revenues obtained from services and goods provided to tourists are distributed among a larger number of people in the community. Thus, tourism is closer to social justice (one of the main pillars of sustainable development) than other sectors of the economy in terms of distributing benefits. On the other hand, travelling to different areas makes societies familiar with the culture and customs of other communities, and provides appropriate conditions for cultural and social interactions which in turn lead to promote peace, security, and promote tolerance among different cultures that are pillars of sustainable development [1–3]. The multidimensional nature of the tourism industry causes major changes in the host society in addition to catering for tourists [4]. Yet, due to the role played by a number of governmental institutions and private sector enterprises, the tourism industry has a very complicated entity [5]. Hence, authorities are attempting to provide the opportunities to take advantage of the positive aspects of this industry by preparing tourism attraction and making them precious in potential areas [6]. One of the pillars of tourism development is the demand for tourism which has a complex structure, because increasing/decreasing contribution of tourism revenues depends on different factors.

Satisfaction of tourists is one of the most important factors that guarantee future profit growth. Nowadays, many organizations have considered tourist satisfaction as an important criterion for measuring the quality of their work. The tourist’s satisfaction is achieved by designing appropriate processes such that services provided meet the expectations of the tourists [7]. Studying of tourism literatures shows that the satisfaction of tourists from a destination/place is an important factor in selecting a destination which means that if the tourists are satisfied with their journey to a destination, they are expected to return or to offer the destination to others. Tourist satisfaction has become a substantial subject for most service industries [8, 9].

Considering that the tourists travel to different areas to achieve mental relaxation, the shortage in quantity/quality of provided services reduces the amount of tourists and prevents the realization of sustainable tourism [10]. Given the importance and increasing contribution of this sector in the modern economy, planning to strengthen the infrastructure of tourism and improving the quality-of-services and facilities for tourists are necessary more than ever.

The city of Hamadan, as an ecotourism and historic destination, can provide a platform for development of sustainable tourism through proper planning and provision of infrastructure. This study aimed a) to identify factors affecting the satisfaction of tourists traveling the city of Hamadan in line with the appropriate decision making for the development of tourism as well as increasing the satisfaction of tourists; and b) to compare performance of two data mining techniques of random forest (RF) and K-nearest neighborhood (KNN) in predicting tourism satisfaction.

## Main text

### Materials and methods

#### Study area

The city of Hamadan is located between latitude 23°59′ and 25° 45′ north and longitude 47° 34′ and 49° 36′ east of the Greenwich Meridian. Hamadan is located in the western part of Iran on the slopes of Alvand mountain range and it has a mountainous weather and has many ancient, historical, natural, cultural, recreational and sportive items for tourist attractions.

Hamadan is the first Persian capital that has been mentioned by Herodotus (the famous Greek historian) [11]. The Ganj Nameh inscriptions (500 BC), the stone lion statue related to of the Medes (the first dynasty of Iran; 728 to 549 BC), the holy tomb of Esther and Mordechai (for the Jews) mentioned in the Torah of the Old Testament, the tomb of Avicenna [the well-known physician and philosopher (about 1000 years ago)], the tomb of Baba Tahir famous poet (about 1000 years ago), the Lalehjin’s Handicrafts city (which was registered as the World Pottery City in UNESCO in 2017), Ali-Sadr Cave, the unique largest water cave in the world, the beautiful Alvand mountain range with the highest peak with an altitude of 3574 meters above sea level and very beautiful valleys, suitable weather, ski resort as well as large sports and recreational complexes are among the tourist areas of Hamadan city [12]. These particular conditions have made Hamadan as a place with a potential for attracting tourists.

#### Statistical Analysis

This study used information of 300 tourists (see Additional file 1 for information about data collection) and utilized two widely used non-parametric methods of RF and KNN to capture any nonlinear relationship between inputs and outputs.

#### Random forest

RF as an ensemble learning method works based on creating several (say 1000) regression trees [13]. In each tree the predictor with smaller prediction error is selected to be at the top of the tree and it is split into two parts (for continuous variable a cutoff point is created using minimizing prediction error). This partitioning continues recursively to create a tree. Prediction is then done using averaging the response variable in each leaves of the final tree (in regression setting). To improve the prediction performance of the tree regression methodology, the random forest technique applies random selection in two ways. In this regard, each tree is formed by using a random sample that is selected from all original observations (bootstrapped sampling), and a random sample of candidate variables (inputs) for splitting [13, 14]. The issue of instability of these trees is handled by these randomness because it leads to introducing differences in individual predictions that are obtained from each tree [15]. In order to obtain predictions for the final forest, the averaging rule is used for the results of all individual trees [14, 15].

#### K-nearest neighborhood

KNN method is one of the most popular non-parametric regression methods. In this method, the distribution function of predictive values is obtained by using a nonparametric distribution of a kernel function. This model predicts future observations based on the similar situation at present, i.e., the probable conditions in the future will be the same as those that occurred at the present time. The probability of occurrence of each state in the current situation depends on the similarity of the observed vector of the independent variables at present and the observed independent vector in the historical series [3].

#### Implementation

To implement the models, variables in Table 1 were used as predictors and TS was used as the output. Then, both RF and K-NN techniques were implemented to identify important variables that affect TS. To provide some goodness of fit measures, we divided the data set into two sets of training and testing (80–20%). The two models were trained using the data in the training set and were tested on the testing set. Evaluation criteria used to investigate the performance of the methods included root mean square error (RMSE), Criterion-referenced measurement (CRM), Nash–Sutcliffe efficiency (NSE) [16], Pearson correlation coefficient (R) and Morgan-Granger-Newbold (MGN) statistic [17].

**Table 1:** Indicators and variables of research

Indicators	Variables
Municipal facilities and equipment	1. Access to urban accommodation centers. 2. Access to station centers for accommodation centers. 3. Access to fuel stations 4. Satisfaction with the status of drinking water in the parks 5. Satisfaction with the status of ATM at the city level 6. Urban furniture 7. Satisfaction with access to rest rooms 8. Satisfaction with city traffic and driving guides. 9. Satisfaction with public transport fleet. 10. Satisfaction with urban traffic. 11. Satisfaction with access to parking. 12. Satisfaction with lighting in the parks and urban spaces
Quality of Environment	1. Climate conditions (Climate comfort); 2. Environmental comfort and the opportunity to rest; 3. Sense of security; 4. Visual and architectural qualities (urban landscape); 5. Satisfaction with pedestrian performance; 6. Satisfaction with urban cleanliness; 7. Satisfaction with city areas; 8. Asphalt pavement of streets and alleys; 9. City entrance scenery; 10. Satisfaction of tourists from the green spaces of the city
Quality of services	1. Quality of souvenirs. 2. Quality of tourist attractions. 3. Quality of entertainment and recreation; 4. Food quality; 5. Design of urban tourism spaces considering the needs of disabled people; 6. Cleaning and maintenance of rest rooms; 7. Satisfaction of tourists with access to health centers and hospitals; 8. Satisfaction with accommodation place; 9. The quality of available Internet
Cost of services	1. Entrance fees and tickets price for tourist destinations; 2. Souvenirs cost; 3. Food costs; 4. Accommodation costs; 5. Transportation costs
Behavior of the host community	1. Honesty of the host community; 2. Satisfaction of tourists with the way citizens addressing; 3. Satisfaction of tourists with the hospitality spirit of citizens; 4. Satisfaction of tourists with citizens’ attitudes towards them; 5. Satisfaction with informing approach in public media in the attraction of tourists; 6. Satisfaction with the way of notification and Installation Symbols Guide to Tourists; 7. Satisfaction of tourists with the guides of housing centers

### Results and discussion

Table 2, shows the demographic characteristics of the individual participated in the study. According to the table, the majority of the participants was male (65.4%), aged between 20 and 45 (54.3%), single (67%) and had academic education (78.4%).

**Table 2:** Characteristics of the Individual Participated in the study

Responsive individual characteristics	Number	Percent
Sex		
Woman	103	34.3
Man	197	65.4
Age		
20–45 year	163	54.3
46–65 year	129	43.3
+ 65 year	7	2.3
Marital status		
Married	111	37
Single	189	67
Education		
High school graduation	65	21.6
Academic education	235	78.4

Both RF and KNN models were optimized and the evaluation criteria were computed over the testing set. Table 3, shows the performance criteria of RF and K-NN. Comparing the results of the two models showed that although RF model produced smaller error rate (RMSE = 0.34) and greater correlation between observed and predicted responses (R = 0.90) compared with the K-NN model, but the K-NN model had smaller biases (CRM = 0.0005) (see Additional file 2: Fig. S1) and skewness of the errors (0.32) (see Additional file 2: Fig. S2). In fact, the precision of the RF model and the bias rate of the K-NN model were better. Accordingly, in order to test the significance difference between the accuracy of the results of the two models, the MGN statistic was used. The value of this statistic was not significant (*P* value = 0.509) indicating that there was no statistical differences between the accuracy of the models.

**Table 3:** Performance criteria of random forest and K-NN

Model	Set	R	RMSE	CRM	NSE	Skewness	MGN
RF	TRAIN	0.94	0.36	0.00024	–	–	RF and K-NN
	TEST	0.90	0.34	− 0.0011	79.46	0.44	0.66
K-NN	TRAIN	0.91	0.39	0.00038	–	–	
	TEST	0.87	0.37	0.0005	74.43	0.32	

Additional file 2: Fig. S3 shows the variable importance (VIMP) for the predictors of satisfaction. As can be seen, society behavior and municipal equipment were the first two top rank variables and cost of services was the least important variable in predicting tourist satisfaction.

### Discussion and conclusion

The tourism industry brings about diversity in economic activities and employment, and realizes the distribution of revenues to a wider range of individuals and groups in societies. In the context of the changes brought about by globalization, only those cities and regions with strategic and futuristic programs have the potential to make optimal use of the advantages of this industry. These cities are mostly creative cities.

The results of this research showed that the behavior of the host community index including the variables such as the honesty of the host community, hospitality spirit, citizens’ attitude towards tourists had the highest effect on the satisfaction of tourists. The way citizens treat tourists reflects the host society’s understanding of tourists in the cultural, social, and economical exchanges. This in turn leads to recognizing the attractions and the financial benefits of tourism as well as to enhancing the conditions for peace and sustainable security among communities. Therefore, the deeper interaction between the host and guest communities in the tourism sector leads to more satisfaction and consequently it results in a thriving in the tourism industry.

The second top most rank variable affecting tourism was municipal facilities and equipment including items such as access to housing centers, access to fuel cell, drinking water, ATMs, urban traffic, etc. This index had a significant impact on tourism with an importance of 0.81. Municipal facilities and equipment are a factor indicating the convenience and comfort as well as the utility of tourist spaces. These factors reduce the hardship of travel and the fatigue caused by displacement and brings pleasure and tranquility to tourists. The evaluation of the outputs of the model indicates the favorable situation that tourists have expressed about the types of urban equipment in Hamadan.

The quality of environment with the importance of 0.488 was the third top most important factor affecting the tourism process. The main elements of this construct are climatic comfort, environmental relaxation, visual quality and urban landscape. In this construct, special attention is paid to the elements of native-oriented tourism. Environmental quality is the underlying factor of paying attention to the protection of natural and human-made environmental identities. The urban landscape reveals the totality of a city as a text, allowing the reader to interpret this text by the interpreter (the tourist). The city of Hamadan has an appropriate environmental quality from the perspective of tourists due to the design and morphology of the city as well as its establishment in the beautiful slopes of Mount Alvand which are among the factors attracting tourists to this city. Other factors involving in the tourism process were the quality of services with coefficient 0.314 and cost of services with coefficient 0.167 that have had less impact on tourism.

Considering the ability and capacity of the ancient city of Hamadan for tourism (which has led to selecting this city as Asian urban tourism pilot and capital in 2018), the need for paying more attention to policies and programs by all policy-makers working in the field of tourism is clear. The consequence of such a broad participation is the continued prosperity of the tourism industry.

## Limitations

One main limitation of this study was the lack of enough international and foreign tourists to compare the results. It is suggested to consider foreign tourists in the future studies.

## Supplementary information

**Additional file 1.** Data collection.

**Additional file 2.** Additional figures.

## Data Availability

The data is available upon the request from the first author.

## Abbreviations

RF: random forest
KNN: K-nearest neighborhood
RMSE: root mean square error
E: determination coefficient
R: Pearson correlation
RMSE: root mean square error
CRM: criterion-referenced measurement
NSE: Nash–Sutcliffe efficiency
MGN: Morgan-Granger-Newbold
