# Supplementary material for: Assessment of factors affecting tourism satisfaction using K-nearest neighborhood and random forest models
Source: BMC Res Notes. 2019 Nov 19;12:749. doi: 10.1186/s13104-019-4799-6 (PMC6862782; doi:10.1186/s13104-019-4799-6)
Supplement: Supplementary file 1 — Additional file 1. Data collection. [file 13104_2019_4799_MOESM1_ESM.docx]

Additional file 1

**Data Collection**

In this study, both documentary sources and survey instruments were utilized in the data collection process. In the former, the factors were extracted and were arranged in a questionnaire. The content validity index (CVR) was used to assess the validity of the questionnaire based on the opinions of the 15 experts (CVR=0.65>0.49) (Lawshe, 1975). Reliability of the questionnaire was evaluated using the Cronbach's alpha which was 0.72. Table 1 shows the effective variables and factors on tourism satisfaction (TS) in Hamadan city. Finally, 300 tourists were selected randomly.

According to the title of the present study, our target population included all tourists that visited Hamadan city. We did not consider any limitation on the gender, language, education, income, etc. to select individuals. Therefore, simple random sampling method was considered to select tourists. To do this, we listed all tourist attraction areas in Hamadan city and attended these areas in different hours of the day during one month. Then, randomly selected tourists were given the questionnaires each day (after they were given a written informed consent) and the data was collected. The questionnaire consisted of 43 items of Likert scale (including 1 = very low, 2 = low, 3 = intermediate, 4 = good and 5 = very good).

.
