# Supplementary material for: Assessment of factors affecting tourism satisfaction using K-nearest neighborhood and random forest models
Source: BMC Res Notes. 2019 Nov 19;12:749. doi: 10.1186/s13104-019-4799-6 (PMC6862782; doi:10.1186/s13104-019-4799-6)
Supplement: Supplementary file 2 — Additional file 2. Additional figures. [file 13104_2019_4799_MOESM2_ESM.docx]

| a)  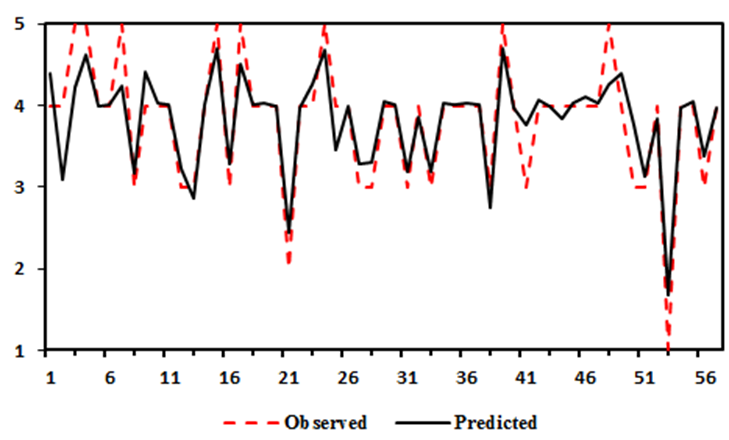 |
| --- |
| b)  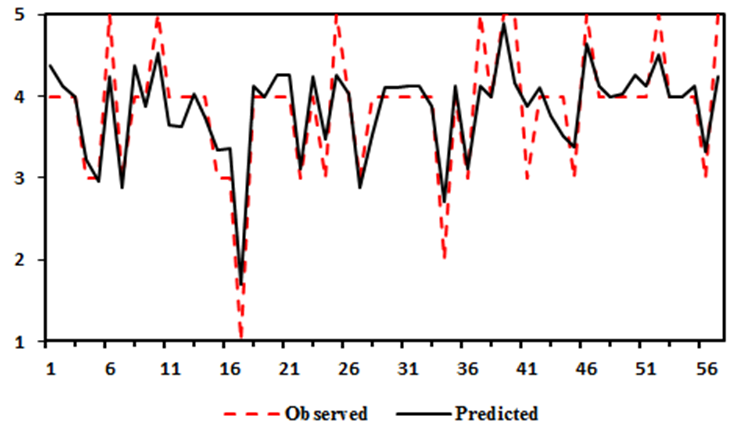 |

Figure S1: Satisfaction prediction values obtained using a) Random Forest and b) K-nearest neighborhood models along with the observed values

| a)  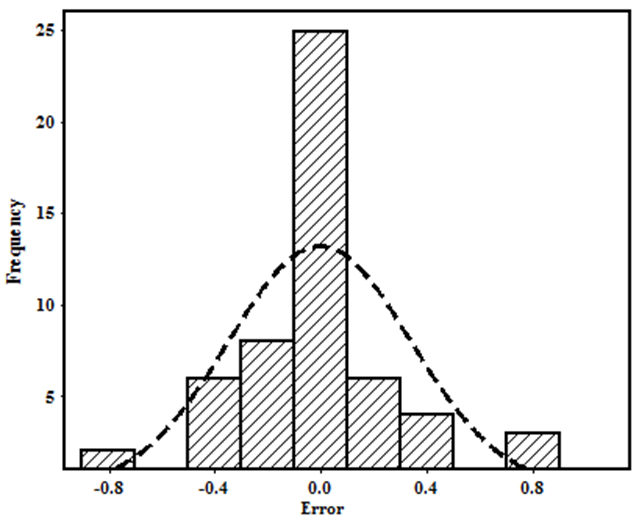 |
| --- |
| b)  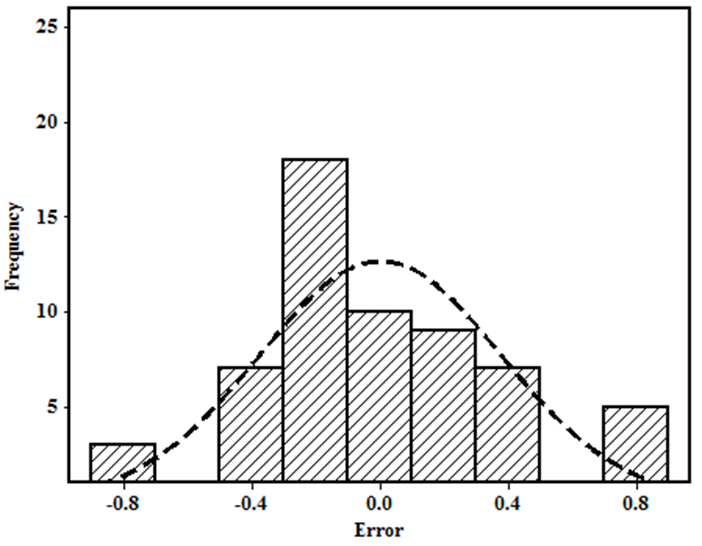 |

Figure S2: The skewness of the predicted values of the response variable obtained by the a) Random Forest and b) K-nearest neighborhood models.


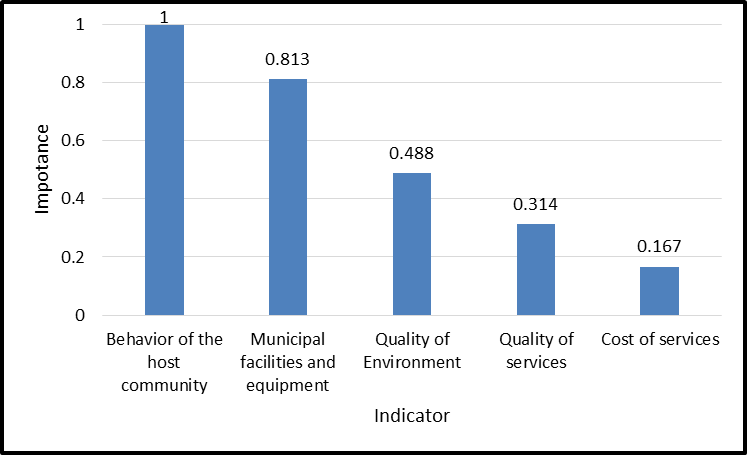


Figure S3: Variable importance of the variables affecting tourism satisfaction
